# Bacterial Adhesion of *Streptococcus mutans* to Dental Material Surfaces

**DOI:** 10.3390/molecules26041152

**Published:** 2021-02-21

**Authors:** Mirjam Kozmos, Petra Virant, Franc Rojko, Anže Abram, Rebeka Rudolf, Peter Raspor, Anamarija Zore, Klemen Bohinc

**Affiliations:** 1 Faculty of Medicine, University of Maribor, Taborska 8, 2000 Maribor, Slovenia; mirjam.kozmos@student.um.si; 2 Faculty of Health Sciences, University of Ljubljana, Zdravstvena 5, 1000 Ljubljana, Slovenia; virant.petra@gmail.com (P.V.); franc.rojko@zf.uni-lj.si (F.R.); anamarija.zore@zf.uni-lj.si (A.Z.); 3 Jozef Stefan Institute, Jamova 39, 1000 Ljubljana, Slovenia; anze.abram@ijs.si; 4 Faculty of Mechanical Engineering, University of Maribor, Smetanova 17, 2000 Maribor, Slovenia; rebeka.rudolf@um.si; 5 Zlatarna Celje d.o.o., Kersnikova 19, 3000 Celje, Slovenia; 6 University of Ljubljana, Kongresni trg 12, 1000 Ljubljana, Slovenia; peter.raspor@guest.arnes.si

**Keywords:** bacterial adhesion, *Streptococcus mutans*, dental materials, surface properties

## Abstract

The aim of this study was to investigate and understand bacterial adhesion to different dental material surfaces like amalgam, Chromasit, an Co-Cr alloy, an IPS InLine ceramic, yttrium stabilized tetragonal polycrystalline zirconia (TPZ), a resin-based composite, an Au-Pt alloy, and a tooth. For all materials, the surface roughness was assessed by profilometry, the surface hydrophobicity was determined by tensiometry, and the zeta potential was measured by electrokinetic phenomena. The arithmetic average roughness was the lowest for the TPZ ceramic (R_a_ = 0.23 µm ± 0.02 µm), while the highest value was observed for the Au-Pt alloy (R_a_ = 0.356 µm ± 0.075 µm). The hydrophobicity was the lowest on the TPZ ceramic and the highest on the Co-Cr alloy. All measured streaming potentials were negative. The most important cause of tooth caries is the bacterium *Streptococcus mutans*, which was chosen for this study. The bacterial adhesion to all material surfaces was determined by scanning electron microscopy. We showed that the lowest bacterial extent was on the amalgam, whereas the greatest extent was on tooth surfaces. In general, measurements showed that surface properties like roughness, hydrophobicity and charge have a significant influence on bacterial adhesion extent. Therefore, dental material development should focus on improving surface characteristics to reduce the risk of secondary caries.

## 1. Introduction

In the last few decades, significant development in dental materials has been observed. In restorative dentistry, established treatment methods have been replaced by advanced methods and techniques [1]. Before new restorative materials are to be clinically implemented, the possible negative impact on human health needs to be clarified [2]. The drawback of the restoration dentistry is a high incidence of secondary caries [3], which is related to restorative materials and bacterial adhesion. A good understanding of bacterial adhesion on dental restorative materials in the oral cavity can be helpful when assessing the etiology of caries [4].

The bacterial fermentation of sugars in food causes tooth demineralization through acid releases [5]. In dentistry, restorative materials are used to replace lost structures [6]. Dental caries in everyday practice is most often found on approximal dental surfaces. Good contact between restoration and tooth structure is therefore of great importance to avoid a high incidence of secondary caries [6,7]. To minimize the risk of secondary caries incidence, it is crucial to have high quality restorative materials that are capable of creating optimal contact between tooth structure and restoration [8].

When contact between restoration and a tooth is compromised, bacteria gain access to protected space where they cannot be mechanically removed; therefore, they can multiply and eventually create biofilm. Generally, the biofilms forming the dental plaque on teeth are composed of several microbial species. One of the prominent bacterial strains that is strongly involved in caries formation is *Streptococcus mutans* (*S. mutans*). While the bacteria initiate lesions in virgin tooth structures in primary caries, in secondary caries, bacterial adhesion takes place in dental restorations [9], particularly at material margins. Material properties greatly affect bacterial adhesion [10], so the optimization of those properties is of great importance to prevent restoration failures. In some cases, indirect materials tend to exhibit better marginal fit, finish, and polish than direct materials [11].

The degradation factors of natural and synthetic materials dictate a proper selection of restorative material [12]. Considering physical and mechanical properties, restorative materials are categorized in four classes: metals, polymers, ceramics, and composites. Metals and their alloys are unique due to their properties and suitability for many dental applications [1]. In dentistry, ceramic materials are used for two major applications: metal-ceramic crowns and fixed partial prostheses. They are also available as orthodontic brackets, dental implant abutments, and ceramic denture teeth [4]. Ceramics have smooth and polished surfaces that are easily cleaned [13]. Resin composites are reinforced polymer materials that can be used for restoring hard tissue, like enamel [14]. The main disadvantage of resin composites is the shrinkage of material [15], which may lead to recurrent decay, hypersensitivity, pulpal inflammation, and restoration failure [16,17].

Bacterial adhesion is a process that is affected by various physico-chemical properties of bacterial and material surfaces [18]. Physico-chemical properties are given by environmental conditions (temperature, nutrition, and acidity), surface properties (roughness, hydrophobicity, and charge), and microorganisms (hydrophobicity, flagellation, and motility) [19,20]. In simple terms, bacterial adhesion is generally described by a two-stage binding model. First, a reversible interaction between the bacterial cell surface and the material surface takes place. The bacterial adhesion is governed by electrostatic, van der Waals, hydrophobic effects, acid–base pairs, and contact interactions [21,22]. The interaction Gibbs free energy of adhesion process shows two minima. The first minimum appears at the separation of few 10 nm and is few kT of energy. Here, the microorganism is weakly and reversibly bound. The second minimum includes specific and nonspecific interactions between so-called adhesion proteins expressed on bacterial surface structures and binding molecules on the material surfaces. Here, the interaction free energy appears at a contact distances of few nm. This means that the microorganism is strongly and irreversibly adhered. The microorganism must surpass a large energy barrier of a few kT to overcome the first minimum and move into the second minimum at the contact.

The surface roughness of dental materials has an important impact on bacterial adhesion and the subsequent biofilm formation [23]. Besides the finishing and polishing of dental restoratives, the composition of dental materials and cleaning agents can also change the roughness of dental materials. Olivera et al. [24] showed that the finishing and polishing of a composite resin significantly decreased the surface roughness of the material. Similar trends were reported by Carneiro et al. [25] for a composite resin with an emphasis on the type of polishing system. They showed that aluminum oxide polishing disk systems promote smoother surfaces compared to impregnated abrasive tips. Zortuk et al. [26] studied the influence of different concentrations of fiber glass on a resin surface roughness and showed that an increasing fiber glass concentration increases the surface roughness of acrylic resin. Further, the cleaning agent used for oral hygiene also influences the roughness of restorative materials. Al-Thobity et al. [27] showed that a cleaning solution significantly increases the surface roughness of different denture resins. Heintze et al. [28] showed that brushing with toothpaste increases the surface roughness of resin composites, whereas ceramic materials showed a significant decrease in surface roughness after the application of toothbrushing.

There are different techniques to determine surface characteristics. Surface roughness can be measured by atomic force microscopy (AFM) or profilometry. AFM allows for the characterization of the surface topography of a material on a submicrometer scale. In the case of surfaces with a roughness greater than dozens of micrometers, mechanical profilometry has emerged as the most reliable technique [29,30,31]. High precision is sacrificed with the use of profilometry, which allows for the characterization of rough materials but lacks resolution [32]. Surface hydrophobicity is measured by tensiometry, whereas zeta potential is measured by electrokinetic technique. SEM is used to directly observe surface-adhered bacteria. Indirect measurements like bacterial staining determine the density of adhered bacteria.

In this study, the bacterial adhesion on dental material surfaces like amalgam, Chromasit, an Co-Cr alloy, an IPS InLine ceramic, a resin-based composite, an Au-Pt alloy, yttrium stabilized tetragonal polycrystalline zirconia (TPZ), and a tooth were examined. Studies [33] have shown that mercury released from amalgam restorations is absorbed and accumulated in various organs such as the kidney, brain, lung, liver, and gastrointestinal tract. Moreover, mercury is capable of crossing through lipid layers at the membrane barriers of the brain and placenta. This fact has become the basis for claims of neuromuscular problems in patients with amalgam restorations, whereas the inorganic form of mercury ions (Hg^+2^) circulates into the blood stream but hardly crosses the blood–brain and placental barriers [11].

Because gold alloys are widely used in dentistry [34], not only for their preferred golden color but also because they maintain an extremely high chemical stability in the mouth, an Au-Pt alloy was selected for testing. This type of Au-Pt alloy also possesses several desirable mechanical properties such as high strength, ductility, and elasticity; moreover, its shows good biocompatibility due to the corrosion resistance of high noble elements [9].

The objective of this research was to investigate the interaction between surface properties and bacterial adhesion extent. Therefore, the surface characteristics of all different materials were measured. Roughness was determined by profilometry, hydrophobicity was determined by contact angle measurements, and zeta potential was determined by measuring the streaming potential. The bacterial adhesion extent was determined from SEM micrographs. For the purpose of our study, *S. mutans*, which is main etiological agent responsible for caries formation and primary colonizing bacteria in the oral cavity, was chosen. *S. mutans* is capable of producing an extracellular polysaccharide matrix that can bind with extracellular glucans (dextran) to a tooth surface. Large amounts of dextran allow *S. mutans* and other bacteria to colonize the enamel and to form cells attached to the teeth as a part of biofilm (dental plaque). The dental plaque protects bacteria against mechanical host-induced forces and may offer some protection against host immune defenses [35].

## 2. Materials and Methods

### 2.1. Bacterial

The *S. mutans* ATCC 25175 strains used in this study were selected from culture on blood agar plates incubated at 37 °C for 48 h with a CO_2_ pack for anaerobic conditions. The *S. mutans* overnight culture was made in a BHI (brain–heart infusion) nutrient broth (Biolife, Italiana Srl) (4012302) at 37 °C for 18 h to obtain a 10^9^ CFU/mL bacterial suspension.

### 2.2. Growth Curve of S. mutans

One milliliter of bacterial suspension (in cell concentration 10^9^ CFU ml^−L^) from overnight culture was taken and diluted to a proximal cell concentration of 10^7^ CFU ml^−L^ in a fresh nutrient broth (BHI broth) and cultivated for 5, 10, 15, 20, and 24 h at 37 °C. The growth of bacteria was measured with pour plate counts on a BHI agar (the standard was ISO 4833-1:2013—the microbiology of the food chain: horizontal method for the enumeration of microorganisms—Part 1: colony count at 30 ℃ by the pour plate technique). Eventually, the inhibitory effect of the tested materials on bacterial growth was tested in parallel aliquots with the presence of tested materials (plates of 100 mm^2^).

### 2.3. Material Surfaces

In this study, eight types of materials most commonly adopted in dental restorative medicine were used and are presented in Table 1 (except for the tooth, which was not changed, plates of 10 mm × 10 mm were prepared). We used these types of metals: an ANA 2000 Duett^®^ amalgam (Nordiska Dental AB, Helsingborg, Sweden), an I-BOND NF (Interdent d.o.o., Celje, Slovenija) Co-Cr alloy, and an Au-Pt alloy (Bioker dental alloy) with a chemical composition of 85.9 wt.% Au, 11.7 wt.% Pt, 1.5 wt.% Zn, Ir, and <1 wt.% In, without Cu and Pd, (Zlatarna Celje, Celje d.o.o., Slovenija). As ceramics, we prepared an IPS InLine ceramic (Ivoclar Vivadent GmbH, Jagst, Germany) and TPZ- DDBioZX2 (Dental Direkt GmbH, Spenge, Germany). Chromasit S1 and D 210/2B (Ivoclar Vivadent GmbH, Jagst, Germany) are a typical representatives of polymers. For the composite, a resin-based composite—the micro-hybrid Z250 composite (3M, St. Paul, MN, USA)—was used.

Cobalt–chrome (Co-Cr) and gold–platinum (Au-Pt) surfaces were cast in a standard size of 10 mm × 10 mm and thickness of 2 mm, respectively. Amalgam plates of size 10 mm × 10 mm and thickness of 2 mm, were prepared using standard pressing techniques: Chromasit with heat-press polymerization, the composite with light polymerization, and ceramic surfaces with sintering (the size and thickness of all materials were 10 mm × 10 mm and 2 mm, respectively).

### 2.4. Surface Characterization

Profilometry was used to characterize the surface topography of the material on a sub-micrometer scale. We used the Form Talysurf Series 2 mechanical profilometer from Taylor-Hobson Ltd., Leicester, Great Britain with a 3 nm resolution in a direction perpendicular to the surface. A 0.8 mm Gaussian filter was applied to distinguish between the roughness and waviness, in accordance with the DIN EN ISO 4288:1998 standard. The contact angle of the materials was measured using a Theta Optical Tensiometer (Attension, Finland) in 5 repetitions. The streaming potential was measured in a 1 mM phosphate-buffered saline (PBS) solution at pH 7.7 and at room temperature with an electrokinetic analyzer (SurPASS, Anton Paar GmbH, Austria). The electrolyte solution was continuously purged with N_2_ after preparation and during measurement to avoid the dissolution of CO_2_ in the solution.

### 2.5. Monitoring the Adhesion Extent

To determine the adhesion of bacteria to dental surfaces, we used the procedure described by Nozaki et al. [36] with some modifications. First, we immersed 10 specimens of each material (expect in the cases of amalgam 4 and TPZ 5) with artificial saliva [37,38] for one hour. Then, we transferred the specimens into a diluted (1:300) overnight culture of *S. mutans* with the BHI broth. Specimens were incubated for 10 h, and, afterwards, the attached bacteria were fixed with 0.1 M PBS. For the time evolution study, we used 5 Chromasit specimens. Bacterial adhesion was determined with SEM via a JEOL JSM-7600F (Tokyo, Japan) microscope. A thin gold layer (7 nm) was applied beforehand to achieve a conductive sample with a GATAN Model 682 PECS system (Precision Ion Etching and Coating System, GATAN Inc., Pleasanton, CA, USA). For quantitative analysis, we manually encircled the bacteria on the SEM micrographs and converted the images to the binary form. We used the ImageJ software package (Version 1.50b, 2015, Wayne Rasband, National Institutes of Health, Bethesda, MD, USA) for further analysis; 110 micrographs, representing a total area of 19,000 µm^2^, were analyzed to determine the coverage of *S. mutans* on the substrates. For the time evolution study, SEM micrographs were prepared separately.

### 2.6. Statistical Analysis

For statistical analysis, the MATLAB software was used. The results of bacterial adhesion extents were compared by Student’s *t*-test at 5% probability level.

## 3. Results

### 3.1. Roughness Measurement

**Figure 1:**
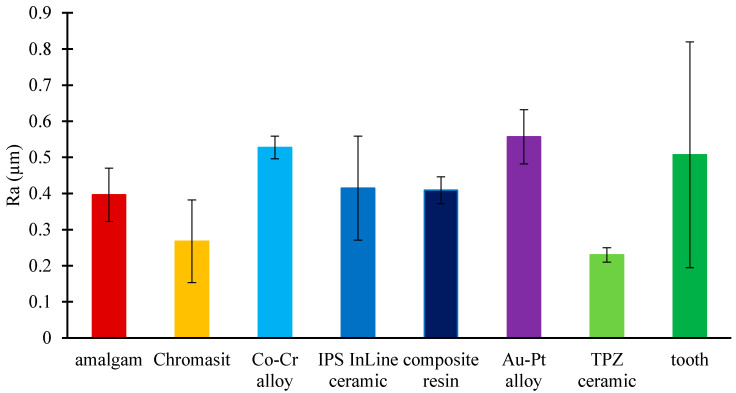
Roughness (Ra) of eight different dental material surfaces: amalgam, Chromasit, Co-Cr alloy, IPS InLine ceramic, resin-based composite, Au-Pt alloy, TPZ ceramic, and tooth.

In the present study, a mechanical profilometer was used to analyze the roughness of all considered dental materials. Figure 1 shows the arithmetic average roughness (Ra) for different dental surfaces. The roughness of the TPZ ceramic (Ra = 0.23 µm ± 0.02 µm) was the lowest, whereas the highest roughness was observed on the Au-Pt alloy surface (Ra = 0.356 µm ± 0.075 µm).

### 3.2. Contact Angle

**Figure 2:**
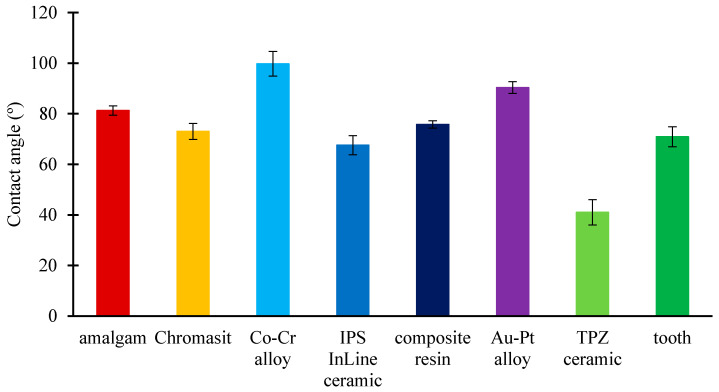
Contact angles of water droplet on dental materials: amalgam, Chromasit, Co-Cr alloy, IPS InLine ceramic, resin-based composite, Au-Pt alloy, TPZ ceramic, and tooth.

With an optical tensiometer, the contact angles of a water droplet on different dental material surfaces were measured. Generally, surfaces with contact angles larger than 90° are considered hydrophobic, whereas surfaces with contact angles smaller than 90° are considered hydrophilic. For each surface type, several measurements were performed, from which the average contact angle and its standard deviation were calculated. Figure 2 shows the contact angles of eight different dental surfaces. The amalgam (81.3°), Chromasit (73.0°), IPS InLine ceramic (67.6°), resin-based composite (75.8°), TPZ ceramic (41.5°), and tooth (70.9°) were found to be hydrophilic surfaces, whereas Au-Pt (90.4°) and Co-Cr (99.7°) alloys were found to be hydrophobic.

### 3.3. Zeta Potential

**Figure 3:**
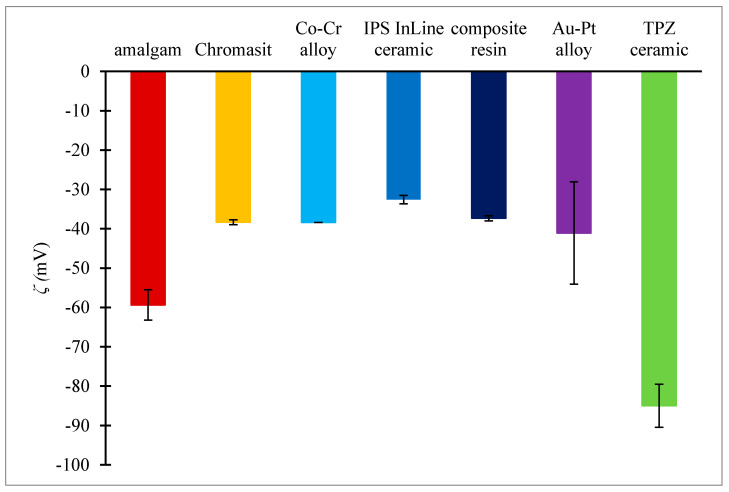
Zeta potentials of seven dental surfaces: amalgam, Chromasit, Co-Cr alloy, IPS InLine ceramic, resin-based composite, Au-Pt alloy, and TPZ ceramic.

The zeta potential measurements (Figure 3) indicated that all dental material surfaces were negatively charged (the potentials were within the range from −85 mV to −32 mV). The most negative zeta potential was observed on the TPZ ceramic (−85 mV ± 5.46 mV). The smallest absolute value of the zeta potential was observed on the IPS InLine ceramic surface (−32.58 mV ± 1.06 mV). The zeta potential measurement on the tooth surfaces could not be performed.

### 3.4. Growth of Bacteria

**Figure 4:**
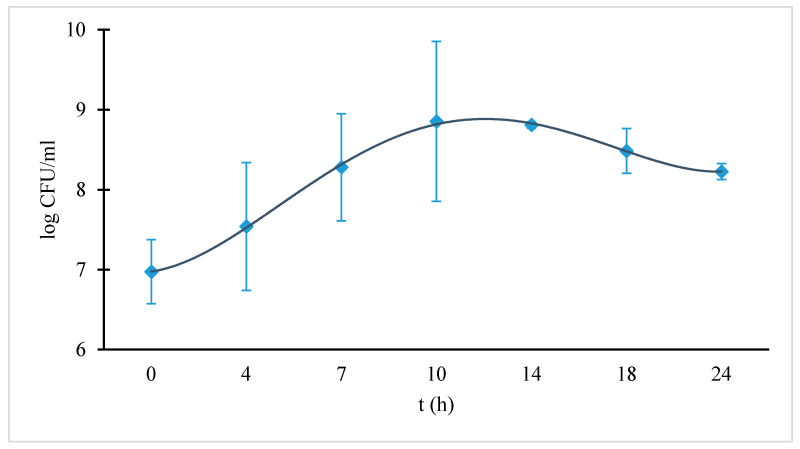
Growth curve of *S. mutans* measured in colony-forming unit per milliliter (CFU/mL) in a BHI (brain–heart infusion) nutrient broth. The diamonds correspond to measured values.

Figure 4 shows the time dependence of the colony-forming units per milliliter. The peak in the bacterial growth was reached after 10 h of incubation at 37 °C. The peak in the curve corresponded to approximately 0.9 × 10^9^ CFU/ml of culture. Due to the lack of nutrients in the broth, the bacterial growth was suppressed after 10 h. The bacterial growth in the BHI medium with the tested materials (amalgam and Au-Pt) was similar to the control bacterial growth without the presence of those materials. After 10 h of incubation at 37 °C, the bacterial concentration reached 10^9^ cells ml^−1^ in the control and tested cultures.

### 3.5. SEM Micrographs

**Figure 5:**
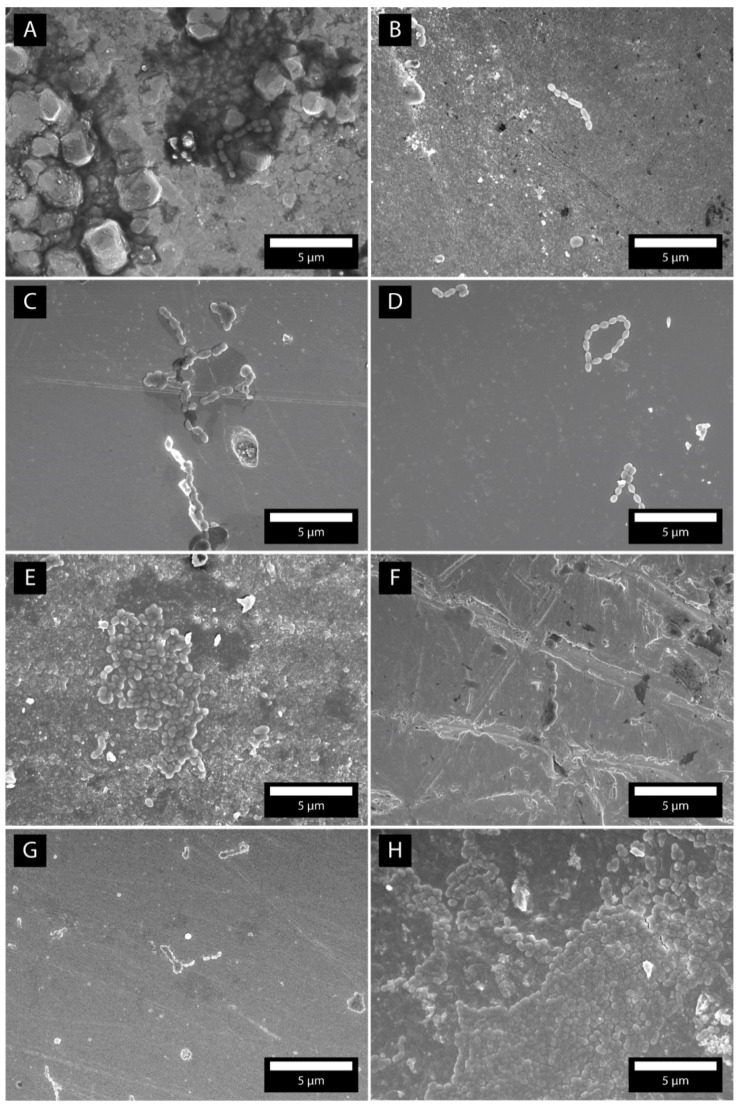
SEM micrographs of the bacteria *S. mutans* after 10 h of incubation time. The micrographs are made on amalgam (**A**), Chromasit (**B**), Co-Cr alloy (**C**), IPS InLine ceramic (**D**), resin-based composite (**E**), Au-Pt alloy (**F**), TPZ ceramic (**G**), and tooth (**H**).

SEM microscopy was used to image the surfaces of the samples and to determine where the bacteria were adhered. Figure 5A–H shows micrographs of dental surfaces with attached bacteria. The images were made after 10 h of incubation. In SEM micrographs, we observed the formation of necklaces (Figure 5C,D,G). On the resin-based composite and tooth surfaces (Figure 5E,H), larger parts of the surfaces were covered with densely packed bacteria. The Au-Pt surface (Figure 5F) was covered with individual bacteria, whereas adhered bacteria were very rarely observed on the amalgam and Chromasit (Figure 5A,B).

### 3.6. Bacterial Adhesion Extent

**Figure 6:**
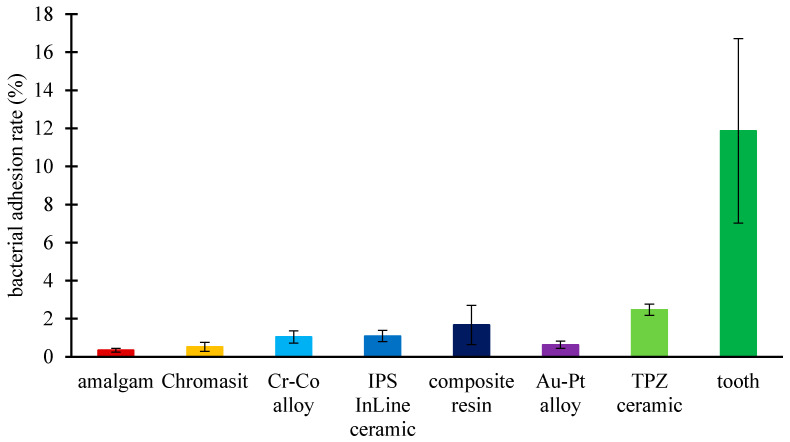
Bacterial adhesion extent on the following dental material surfaces after 10 h incubation: amalgam, Chromasit, Co-Cr alloy, IPS InLine ceramic, resin-based composite, Au-Pt alloy, TPZ ceramic, and tooth. The *t*-test analyses for bacterial extents are as follows. Amalgam/Chromasit: *p* = 0.0025; amalgam/Au-Pt alloy: *p* = 0.00001; amalgam/Co-Cr alloy, IPS InLine ceramic, composite resin, TPZ ceramic, and tooth: *p* < 10^−5^.

The bacterial adhesion of the dental surfaces that were incubated for 10 h was evaluated from a series of SEM micrographs (Figure 5). Figure 6 shows the bacterial adhesion extent, which is defined as a ratio between the surface area covered by bacteria and the full surface area of the material. We observed a minor bacterial adhesion extent on the amalgam (0.346%) and Chromasit (0.519%). In contrast, we observed a large bacterial adhesion extent on the TPZ ceramic (2.47%) and tooth surfaces (11.87%). A *t*-test analysis showed that the bacterial adhesion extent on the amalgam was significantly smaller than the extent on the Au-Pt alloy (*p* = 0.00001) and Chromasit (*p* = 0.0025). The bacterial adhesion extent on the amalgam was statistically significant compared to all other materials (the Co-Cr alloy, the IPS InLine ceramic, the composite resin, the TPZ ceramic, and the tooth) with *p* < 10^−5^.

### 3.7. Time Evolution of Bacterial Extent

**Figure 7:**
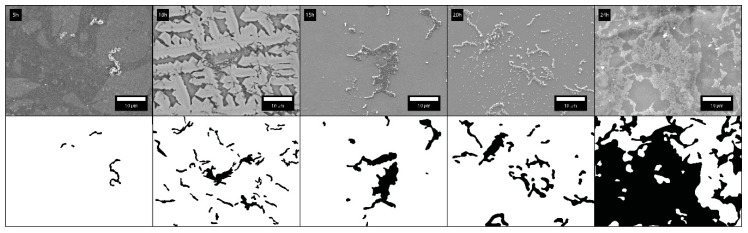
Time evolution of adhered bacteria on Chromasit (5, 10, 15, 20, and 24 h).

The bacterial adhesion on dental surfaces is time-dependent. Figure 7 shows SEM micrographs for five different incubation times (5, 10, 15, 20, and 24 h). The quantification of bacterial adhesion was represented by the surface coverage, which is defined as a percentage of the surface area covered by bacteria (Table 2). We observed that after 20 h, biofilm formation started, whereas the adhesion of bacterial chains was observed before 20 h.

### 3.8. Different Materials in Contact

**Figure 8:**
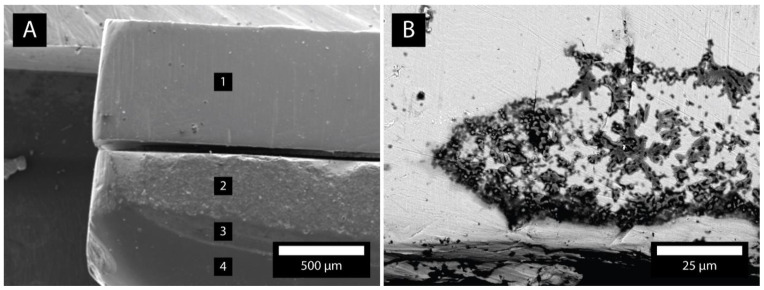
(**A**) Plates composed of Ti (1), Au (2), and the composite (3 and 4). (**B**) Gap between materials 1 and 2 with adhered bacteria.

**Figure 9:**
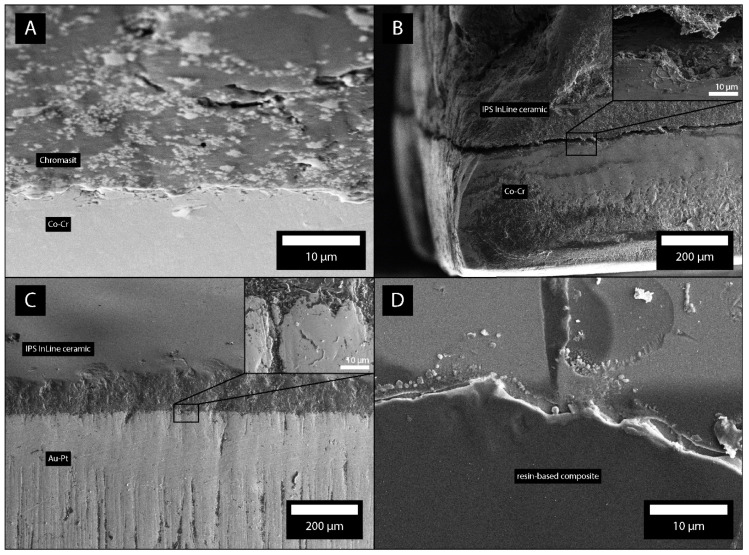
Plates composed of Chromasit and Co-Cr (**A**), ceramics and Co-Cr (**B**), ceramics and Au-Pt (**C**), and composite (**D**). The gaps between the materials with adhered bacteria are shown by SEM. The insets show the regions of pronounced bacterial adhesion.

In our study, we also considered bacterial adhesion on different materials in contact. Figure 8A shows the plates of Ti, Au, and the composite in contact. The SEM micrographs show that most bacteria adhered to the gap between the contacts of materials (Figure 8B). The flat parts of materials possessed much less bacteria. Figure 9A shows the plates of Chromasit and Co-Cr in contact. Figure 9B,C show the contact of ceramic with Co-Cr and Au-Pt alloys, respectively. The most bacteria are adhered inside the gap between the materials. Figure 9D shows the resin-based composite with adhered bacteria.

## 4. Discussion

Dental amalgam has been used in dentistry for 150 years due to its inexpensiveness, bacteriostatic effect, elastic properties, and ease of use [39]. It is an excellent restorative material, but its toxicity due to the content of mercury has always raised concerns. Due to its ubiquitous presence, humans are routinely exposed to small amount of mercury from the air, drinking water, and diet [15]. Amalgam restorations continuously release mercury into the oral cavity, but there is no clear evidence that this has an impact on human health [40]. Some individuals develop adverse hypersensitivity reactions on amalgam, and these can be seen as dermatological or oral symptoms and usually disappear in a few days after the removal of these restorations [41]. The increasing demand for aesthetics coupled with mercury concerns have led to the development of highly aesthetic, resin-based composites as restorative materials [42,43].

In a seven-year follow-up study on amalgam and composite restorations, Bernardo et al. [44] reported that the main reason for restoration failure over time is secondary caries, usually formed at the contact between the restoration and the tooth. In their study, Ionescu et al. [43] demonstrated that *S. mutans* biofilm formation was significantly reduced by silver-polysaccharide antimicrobial nanocomposite coatings. Therefore, the interest for similar anti-adhesive and antimicrobial coatings, as well as antibiotic-release systems, is increasing [43,45].

Bacterial adhesion is affected by various physico-chemical properties of bacterial and material surfaces. In this study, the impact of material properties such as surfaces roughness, charge, and hydrophobicity on the adhesion potential of *S. mutans* was investigated. Dental materials that are most commonly used in dental practice were chosen. The surface roughness of dental materials was determined by a profilometer, while the surface charge was determined by zeta potential measurements. For all surfaces, the contact angles with water were measured by tensiometer. In final stage, bacterial adhesion on dental materials was determined by SEM.

In the first part of the present study, surface characteristics, which were found to have an influence on the bacterial adhesion, were investigated. The literature states that increasing surface roughness also increases adhesion [44,45,46,47,48]. Among tested materials, the bacterial adhesion on the Au-Pt plate (Ra = 0.356 μm ± 0.0070 μm) remained low despite the highest measured roughness. The results of roughness obtained by the profilometer were consistent with the findings of Hahnel et al. [49], who demonstrated that the surface roughness of different ceramic materials has no significant influence on bacterial adhesion. A characteristic that has a significant effect on bacterial adhesion is surface charge. All measured material surfaces showed negative zeta potential values and a low adhesion extent, which was consistent with study performed by Song et al. [50], who reported that bacterial adhesion is higher on positively-charged rather than negatively-charged surfaces. A property that plays and important role in initial bacterial adhesion is hydrophobicity, which depends on bacterial and material surfaces [51]. Olivera et al. [52] showed that the interaction between hydrophobic bacteria and hydrophobic surfaces leads to higher levels of bacterial adhesion. In this study, we found that the Co-Cr and Au-Pt alloys surfaces were slightly hydrophobic (99.74° ± 4.88° and 90.35° ± 2.32°, respectively), but the level of adhesion remained low.

In the second part, the adhesion extent of *S. mutans*, which is a primary etiological agent in caries initiation, was observed [53]. In our research, among tested dental materials, amalgam (0.346%) showed the lowest adhesion level of *S. mutans*. This might have been due to its mercury, which seemed to have adverse effect on the growth extent of *S. mutans*. On the other hand, the highest adhesion level of *S. mutans* was observed on the TPZ surface and the tooth (2.47% and 11.87%, respectively), despite the highest negative value of zeta potential (−85 mV ± 5.46 mV) and the lowest surface roughness (Ra = 0.23 µm ± 0.02 µm). For further material development and better adhesion extent understanding, correlations between those characteristics need to be investigated.

Bacterial adhesion is the initial step in biofilm formation and the main reason for two of the most prevalent and globally ubiquitous diseases, namely dental caries and periodontal diseases [54]. Dental biofilm formation starts a few seconds after cleaning the tooth surface via the colonization of early colonizing microbiota, mostly streptococcal species [55]. With the maturation of dental biofilm, the composition of bacteria in the biofilm changes from a Gram-positive to Gram-negative bacteria [54]. The maturation and dysbiosis of oral microbiota induce the development of gingivitis and periodontitis [56]. Scientific research has shown implications of oral diseases in the development of different systemic pathologies. A literature reviewed by Fiorillo et al. [57] focused on the correlations between oral health and systemic diseases, and it concluded that periodontal disease could be a further cause of system pathologies. They showed that oral microbiota and periodontal disease can have influence on cardiovascular diseases, rheumatoid arthritis, and neurodegenerative pathologies. Therefore, a full understanding of oral biofilm complexity can be a good starting point in the development of preventive techniques that are oriented to inhibit the formation of biofilm and to achieve good general health.

The bacterial adhesion extent depends on roughness, material surface streaming potential, and hydrophobicity, as well as bacteria surface properties. The examined characteristics of materials should therefore be considered in new dental material development to reduce bacterial adhesion and secondary caries formation. A promising way to reduce the risk of restoration failure is the use of contact-active dental materials, which are able to interact with oral microflora. Since the field of contact-active dental materials that can lower the bacteria adherence extent is highly auspicious, this study followed these trends. Further studies to determine which composites and materials can comply with all above-mentioned factors need to be conducted.

## 5. Conclusions

In this study, we examined the influence of dental surface characteristics on the adhesion of *S. mutans*. The surface topography of different dental materials was determined by profilometry, the contact angle of the materials was measured by tensiometry, and the zeta potential was determined by electro-kinetic measurements. From the SEM micrographs, we determined the bacterial adhesion extent. The results demonstrated that surface characteristics and the extent of bacterial adhesion have a positive correlation. This experimental study helps to understand which restoration material can reduce bacterial adhesion when exposed to the oral environment. The following main conclusions can be drawn from this study:

- We indicated that the TPZ ceramic had the lowest determined roughness, whereas the highest roughness was observed on the Au-Pt alloy surface. Amalgam, Chromasit, the IPS InLine ceramic, the resin-based composite, the TPZ ceramic, and the tooth were hydrophilic, whereas the Au-Pt and Cr-Co alloys were hydrophobic. The zeta potential measurements indicated that all tested dental material surfaces were negatively charged.
- The bacterial growth in the BHI medium with the tested materials showed that amalgam is the optimal surface regarding bacterial adhesion.
- SEM observations revealed that on the resin-based composite and tooth surfaces, a larger part of the surfaces was covered with densely packed bacteria. The Au-Pt surface was covered with individual bacteria, whereas adhered bacteria were very rarely observed on the amalgam and Chromasit. Correlations between those characteristics need to be investigated for further material development and better adhesion rate understanding.

The lowest bacterial adhesion extent of *S. mutans* was observed for the amalgam. The bacterial adhesion extent on the amalgam, Chromasit, and the Au-Pt alloy was much lower than the adhesion extent on the tooth. Considering non-toxic and non-corrosion properties of the Au-Pt alloy is the optimal material regarding health. On the other hand, amalgam is much cheaper and has very good gap sealing properties.

The preparation of optimal dental materials demands the control of their physical, biological, and microbiological characteristics. In the further development of new dental materials, it will be necessary to consider the studied characteristics with the aim of reducing bacterial adhesion and the formation of secondary caries. A promising way to reduce the risk of restoration failure is the use of contact-active dental materials that can interact with oral microflora so that a minimum level of bacterial adhesion is achieved. Here, we mention nano-structured platforms based on calcium phosphate and metallic particles, which have shown the prevention of the mineral loss of a hard tooth structure and protection against caries-related pathogens. Certainly, research on these novel materials are needed to confirm a real breakthrough in the longevity of restorative dental materials.

## Figures and Tables

**Table 1:** Materials most commonly used in dental practice sorted by its composition into 4 groups. TPZ: tetragonal polycrystalline zirconia.

Materials for Dental Application			
Metals	Polymers	Ceramics	Composites
Amalgam	Chromasit	IPS InLine ceramic	Resin-based composite
Co-Cr alloy		TPZ ceramic	
Au-Pt alloy			

**Table 2:** Time evolution of adhered bacteria to the Chromasit surface. Surface coverage corresponds to the percentage of the surface area covered by bacteria.

Time Evolution of Bacterial Extent on the Chromasit Surface					
**Time**	5 h	10 h	15 h	20 h	24 h
**Surface Coverage**	0.76%	7.71%	7.13%	9.01%	61.8%

## Data Availability

Data is contained within the article.

## Sample Availability

Samples of the compounds are not available from the authors.
